# Sex‐specific diet differences in harbor seals (*Phoca vitulina*) via spatial assortment

**DOI:** 10.1002/ece3.11417

**Published:** 2024-07-03

**Authors:** Holland C. Conwell, Zoë K. Lewis, Austen Thomas, Alejandro Acevedo‐Gutiérrez, Dietmar Schwarz

**Affiliations:** ^1^ Biology Department Western Washington University Bellingham Washington USA; ^2^ Smith‐Root Vancouver Washington USA

**Keywords:** diet analysis, DNA metabarcoding, marine mammals, predator–prey interactions, sex identification, sites of conservation concern

## Abstract

The lack of recovery of Chinook salmon (*Oncorhynchus tshawytscha*) in the Pacific Northwest has been blamed in part on predation by pinnipeds, particularly the harbor seal (*Phoca vitulina*). Previous work at a limited number of locations has shown that male seal diet contains more salmon than that of female seals and that sex ratios at haul‐out sites differ spatiotemporally. This intrapopulation variation in predation may result in greater effects on salmon than suggested by models assuming equal spatial distribution and diet proportion. To address the generality of these patterns, we examined the sex ratios and diet of male and female harbor seals from 13 haul‐out sites in the inland waters of Washington State and the province of British Columbia during 2012–2018. DNA metabarcoding was conducted to determine prey species proportions of individual scat samples. The sex of harbor seals was then determined from each scat matrix sample with the use of quantitative polymerase chain reaction (qPCR). We analyzed 2405 harbor seal scat samples using generalized linear mixed models (GLMMs) to examine the factors influencing harbor seal sex ratio at haul‐out sites and permutational multivariate analysis of variance (PERMANOVA) to examine the influence of sex and haul‐out site on harbor seal diet composition. We found that the overall sex ratio was 1:1.02 (female:male) with notable spatiotemporal variation. Salmoniformes were about 2.6 times more abundant in the diet of males than in the diet of females, and Chinook salmon comprised ca. three times more of the average male harbor seal's diet than the average female's diet. Based on site‐specific sex ratios and diet data, we identified three haul‐out sites where Chinook salmon appear to be under high predation pressure by male harbor seals: Cowichan Bay, Cutts Area, and Fraser River. Our study indicates that combining sex‐specific pinniped diet data with the sex ratio of haul‐out sites can help identify priority sites of conservation concern.

## Graphical abstract

Our analysis highlights the variation in male and female harbor seal distribution and diet at a relatively large scale in the Salish Sea (the marine inland waters of Washington State, USA, and British Columbia, Canada). Diet differences between males and females appear to be largely the effect of sex‐specific spatial assortment combined with opportunistic use of local prey resources. A previously reported male harbor seal bias for salmon appears to extend across the Salish Sea, and salmon runs at male‐dominated haul‐out sites may experience disproportionate predation pressure.

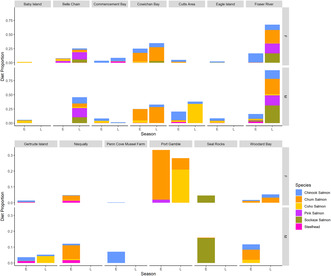

## 1 INTRODUCTION

Predation has a direct effect on prey abundance in any ecosystem (Hairston Jr. & Hairston Sr., 1993; Menge & Sutherland, 1976). The impact of this predation on specific prey species varies depending on whether predators specialize in the prey species at hand or are generalists in their ecosystems (Hanski et al., 1991; Jiang & Morin, 2005). However, some prey specialization studies have found that populations of predators that are widely accepted to be generalists can be composed of many individual specialists or even groups of individual specialists (Bolnick et al., 2003; de Lima et al., 2019). In the case of marine mammals, numerous studies have revealed varying diet specialization or diet differences between sexes (Elliott Smith et al., 2015; Estes et al., 2003; Louis et al., 2022; Riverón et al., 2021). Initial studies in the Salish Sea (the marine inland waters of Washington State, USA, and British Columbia, Canada) indicate diet specialization and intrapopulation feeding diversity between males and female harbor seals (*Phoca vitulina*), possibly resulting in differential impacts on prey species of conservation concern (Schwarz et al., 2018; Voelker et al., 2020). Although stable for the past two decades, harbor seal numbers in the region climbed for several years after the implementation of the United States Marine Mammal Protection Act in 1972 (Jefferson et al., 2021; Jeffries et al., 2003). Consequently, concerns have arisen about their predation impact on Pacific salmon (*Oncorhynchus* spp.)—hereafter salmon (Chasco et al., 2017; Scordino, 2010). Within Washington State, Chinook salmon are listed as threatened and endangered, sockeye salmon (*Oncorhynchus nerka*) as endangered, and coho (*Oncorhynchus kisutch*) and chum (*Oncorhynchus keta*) salmon are considered threatened (Endangered and Threatened Wildlife, 2014, 2016). Another species of conservation concern, steelhead (*Oncorhynchus mykiss*) is threatened in parts of Washington State (Endangered and Threatened Wildlife, 2016). The proportion of salmon in the diets of male and female harbor seals in the region has been estimated before; however, the findings came from only two estuarine haul‐out sites in the Strait of Georgia, Canada (Schwarz et al., 2018). It is thus unknown if these sex‐specific diet differences are limited to these two sites or if they apply across the Salish Sea.

Salmon are important to the cultural identity and traditions of the Coast Salish Indigenous Peoples and have massive economic influence through lucrative commercial and recreational fishing (TCW Economics, 2008). Further, Chinook salmon (*O. tshawytscha*) in the Salish Sea is critical to Southern Resident killer whales (*Orcinus orca*)—an iconic yet endangered population (Hanson et al., 2021). Salmon stocks have declined over the last century, in part due to habitat loss and degradation, environmental fluctuations, and harvesting pressure (Lichatowich et al., 1999; Nehlsen et al., 1991; Sobocinski et al., 2021). While not one of the major contributing factors in the initial decline, harbor seal predation may be hindering the recovery of salmon stocks (Chasco et al., 2017; Sobocinski et al., 2021). Worldwide, harbor seals are viewed as generalist predators with seasonal and regional differences in diet (Burns, 2009). However, harbor seals in the Salish Sea appear to consist of a generalist population with strong variation in individual diet (Bjorkland et al., 2015; Bromaghin et al., 2013; Howard et al., 2013; Lance et al., 2012). Schwarz et al. (2018) examined harbor seal diet by sex at Comox and Cowichan Bay in the Strait of Georgia, British Columbia, Canada, and found a male dietary bias for adult salmon and a female bias for predators of young salmon. In addition, the ecological effects of a male harbor seal bias for salmon could be further compounded if males are more concentrated in locations critical for threatened salmonids. Schwarz et al. (2018) found spatiotemporal variation in harbor seal sex ratios in the Salish Sea, leaving the potential for some salmon stocks to be more heavily impacted than others. Yet, the extent of this spatiotemporal variation in harbor seal sex ratios and sex‐specific dietary biases in the Salish Sea is unknown. Based on the findings of Schwarz et al. (2018), we anticipated differences in sex ratios and sex‐specific harbor seal diet applicable to the larger Salish Sea region.

Here, we describe the sex ratios and diet of male and female harbor seals from different haul‐out sites and/or years than those documented by Schwarz et al. (2018) with the goal of contributing to informed management decisions. To investigate sex‐specific dietary differences, sex ratios, and the potential impact of both on threatened salmon, we processed harbor seal scat collected between 2012 and 2018 from 13 haul‐out sites across the Salish Sea and analyzed scat samples with a combination of DNA metabarcoding of prey species and molecular sex identification of the depositing seals via quantitative polymerase chain reaction (qPCR).

## 2 METHODS

### 2.1 Collection and selection of harbor seal scat

**FIGURE 1:**
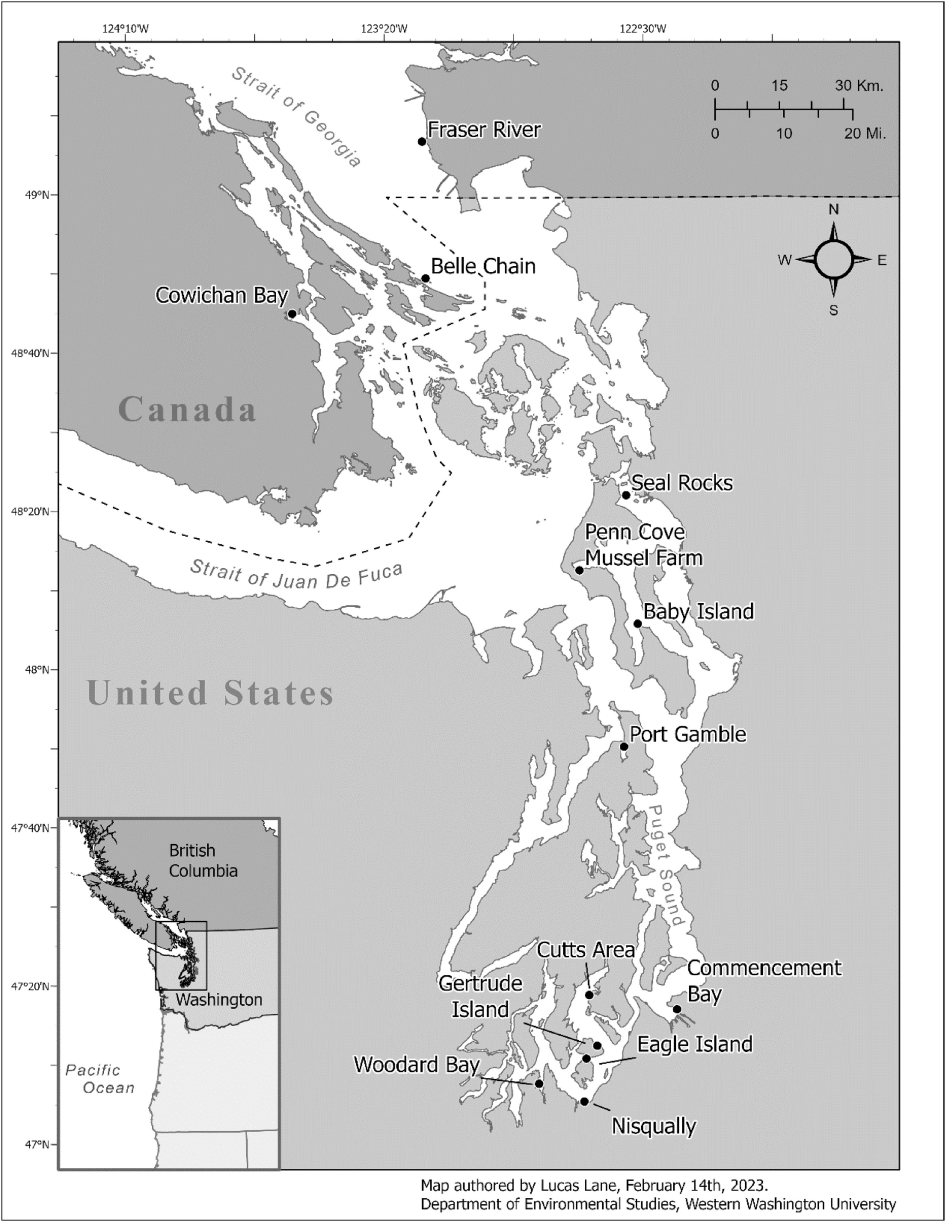
The Salish Sea region where harbor seal scat was collected. Black dots indicate the 13 scat collection haul‐out sites included in data analysis.

Harbor seal scat samples used in this study were a subset of scat samples collected from two studies spanning a total of 56 haul‐out sites across the Salish Sea from 2011 through 2019 between northern Georgia Strait and Puget Sound (Thomas et al., 2022; Voelker et al., 2020). We conducted quantitative polymerase chain reaction (qPCR) analysis to determine the sex of the harbor seal depositor for a subsample of 15 haul‐out sites from Thomas et al. (2022) and combined these results with the sexing results from all five different haul‐out sites in Voelker et al. (2020). Out of the 20 haul‐out sites from the two studies, we did not include haul‐out sites and/or years that were previously analyzed in Schwarz et al. (2018) (thereby excluding Comox 2012–2013 and Cowichan Bay 2012–2013). We also removed six other haul‐out sites from analysis. Five of these haul‐out sites were excluded for having <25 samples, and one site, Baby Island, was a duplicate in both diet data sets. Thus, we ended up with diet and sex data from 13 haul‐out sites across the Salish Sea from 2012 to 2018 (Figure 1). Out of these 13 haul‐out sites, nine sites had >100 samples—hereafter referred to as the “well‐sampled” sites to distinguish them from sites with fewer samples that may be less representative of seal diet. These nine well‐sampled sites represent ca. 15% of haul‐outs with >100 individuals in the area between our northern‐ and southernmost sample locations and have been in use for at least 25 years (Jeffries et al., 2000).

Scat collection followed a standardized protocol described by Thomas et al. (2014, 2022). Scat samples were collected using disposable wooden tongue depressors and stored in individual 500 mL plastic histology containers (or zip‐style bags) lined with 126 μm nylon mesh paint strainers. The samples were either preserved onsite with the addition of 300 mL of 95% ethanol to the sampling containers or frozen in the lab at −20°C within 6 h of collection. At the time of processing, scat samples were thawed, and sampling containers were filled with 95% ethanol. Hard prey remains isolated from the scat matrix through manual homogenization of the paint strainers containing thawed scat samples. Once only isolated hard prey remained in the paint strainers, the strainers were removed from the ethanol‐preserved scat matrix contained in the histology containers. Subsequently, the paint strainers enclosing hard prey remains and the histology containers containing preserved scat matrix material were refrozen until the time of analysis.

### 2.2 Determination of harbor seal diet

Prior to analysis, preserved scat matrix material was subsampled, centrifuged, and dried until all ethanol was removed (Thomas et al., 2022). DNA was then extracted from the dried sample using the QIAGEN QIAamp DNA Stool Mini Kit in accordance with manufacturer protocols. Subsequent DNA metabarcoding analysis yielded the proportion of prey species found in each scat sample used to describe harbor seal diets (Thomas et al., 2017). A 16S mtDNA fragment (~260 bp), which varies among fish and cephalopod species, was used as a metabarcoding marker in DNA metabarcoding analysis to determine which prey species were present in harbor seal scat samples (Thomas et al., 2022). Extracted DNA was amplified through PCR. A secondary reaction using the cytochrome oxidase I (CO1) DNA barcode region was necessary since the initial 16S marker could not differentiate between coho salmon and steelhead DNA sequences (Thomas et al., 2022). Scat sample amplicons were prepared for sequencing using the Illumina TruSeqTM DNA sample prep kit, which was then completed on Illumina MiSeq using the MiSeq Reagent Kit v2 (300 cycles) for SE 300 bp reads (Thomas et al., 2022). Prey species were then indexed using nucleotide BLAST and a custom reference library of fish and cephalopod DNA sequences (Thomas et al., 2022). The proportions of DNA barcoding reads for different prey species in individual harbor seal scat samples were described as Relative Read Abundance (RRA, Thomas et al., 2022) and are referred to in the following as “diet proportions” for convenience. These proportions likely represent biased estimates of the actual seal diet (Thomas et al., 2014), but they should nevertheless be useful given the comparative focus of our study.

### 2.3 Determination of harbor seal sexes

To determine the sex of harbor seals from the collected scat samples, we used quantitative polymerase chain reaction (qPCR). The qPCR procedure used in this study was modified from the protocol developed by Matejusová et al. (2013), as described in Schwarz et al. (2018) and Voelker et al. (2020). Each sample underwent two Taqman qPCR reactions that targeted the paralogous zinc finger x (ZFX) and zinc finger y (ZFY) genes. At least two ZFX and two ZFY replicate qPCR reactions were run for every sample. Samples were classified as male if at least one ZFX and ZFY replicate showed amplification. Samples were conversely classified as female if at least one ZFX replicate was amplified, but no amplification occurred in either ZFY reaction. Samples were rejected altogether if amplification failed to occur in both ZFX reactions. Each 96‐well test plate contained DNA from known male and female harbor seals in captivity, which served as positive controls. Each 96‐well test plate additionally included negative controls that did not contain template DNA. We successfully sexed 73% of the samples that underwent qPCR analysis. We then estimated the approximate false negative rate for cases in which both ZFY replicates are expected to fail for true males using a formula previously applied in Schwarz et al. (2018) and Voelker et al. (2020). We found this approximate false negative rate to be 0.29%, which was lower than the false negative rates from Schwarz et al. (2018) and Voelker et al. (2020). Before applying the formula to calculate the false negative rate for true males, we excluded samples with any ZFX reactions that failed to amplify. Samples that were excluded from this calculation were still included in the data analysis.

### 2.4 Data analysis

To examine the factors influencing harbor seal sex ratio, we used generalized linear mixed models (GLMMs) with maximum likelihood estimation and Gauss‐Hermite Quadrature in R (R Core Team, 2022). Using the package lme4, we then compared GLMMs using binomial error with logit transformation (Bates et al., 2015). Factors included in the GLMMs as fixed effects were haul‐out site and season (early = January–June or late = July–December) while the year was treated as a random effect. The division of samples into early and late season follows Schwarz et al. (2018) and roughly mirrors the division of salmon life history into juvenile outmigration and adult return. For all linear modeling, models with the lowest AIC values were considered to be the best models, and marginal and conditional $R^{2}$ values were reported (Tables 2 and 5; Nakagawa & Schielzeth, 2012).

Using the diversity function in R package vegan, we generated Shannon diversity indices to assess the influence of sex, haul‐out site, and haul‐out site type on diet diversity at the well‐sampled sites (Oksanen et al., 2022). We pooled diet data by prey order and averaged diet proportions for all 22 prey order groups by haul‐out site, sex, haul‐out site type, year, and month. The Embiotocidae family (Ovalentaria *incertae sedis*) was treated as a separate group and compared alongside prey orders. We then used linear mixed‐effects models (LMM) with maximum likelihood estimation in R to examine the factors influencing Shannon diversity indices (R Core Team, 2022). We included sex, haul‐out site, and haul‐out site type as fixed effects and season and year as random effects and used the package lme4 to run LMMs (Bates et al., 2015). To characterize male and female harbor seal diet, we calculated the average of all DNA diet fractions (RRA values) for each given prey taxon for samples classified as male or female, respectively.

We used permutational multivariate analysis of variance (PERMANOVA) to examine the influence of sex and haul‐out site on harbor seal diet composition. Following Schwarz et al. (2018), we first pooled diet data by prey order and averaged diet proportions for each order by haul‐out site, year, month, and sex. These averages encompassed all orders with an average diet proportion, or mean RRA value, across the whole dataset of >0.01, which resulted in a group of ten prey orders for this analysis. Using R package vegan, we applied the betadisper function to test for overdispersion of the Bray–Curtis distances (Oksanen et al., 2022). We then applied the adonis2 function in R package vegan to conduct a PERMANOVA using Bray–Curtis distances with 999 permutations (Oksanen et al., 2022). We tested for the significance of sex with the whole dataset. We then subdivided the dataset by season and tested for sex, haul‐out site, and the interaction for both sex and haul‐out site for early and late season separately.

The harbor seal scat samples analyzed in this study were collected from January to November in the early and late seasons (Table 1). Since this study is a meta‐analysis of harbor seal diet, the samples analyzed here were not evenly distributed among years and defined seasons. Some haul‐out sites were better sampled than others and some sites were only sampled during specific years and seasons (Table 1). Belle Chain, Cowichan Bay, and Fraser River had the most scat collected in the late season but little in the early season (Table 1). These three haul‐out sites were additionally only sampled from 2012 through 2014 (Table 1). The remaining ten haul‐out sites had better sampling coverage in the early season but were only sampled from 2016 through 2018 (Table 1). Because this spatiotemporally uneven sampling arose concerns about season and year acting as a proxy for site and geographic region, season and year were treated as random effects in GLMMs and LMMs. The data were also subdivided by season before conducting PERMANOVA to remove this factor from the results.

**TABLE 1:** Number of harbor seal scat samples collected in the Salish Sea from 13 haul‐out sites over the early and late seasons of 2012 through 2018 for which sex and diet were successfully determined.

Haul‐out site	Year	2012		2013		2014		2016		2017		2018		Subtotal
	Season	E	L	E	L	E	L	E	L	E	L	E	L	
Baby Island								182	11					193
Belle Chain		7	76	0	52									135
Commencement Bay								0	99			207	0	306
Cowichan Bay						29	103							132
Cutts Area								53	4	52	0	80	0	189
Eagle Island								59	5	46	0	8	0	118
Fraser River		33	91	36	55									215
Gertrude Island								167	46	170	0	47	0	430
Nisqually						20	0	65	0	17	0	134	0	236
Penn Cove Mussel Farm								50	7					57
Port Gamble								4	39					43
Seal Rocks								40	1					41
Woodard Bay								223	29	4	0	54	0	310
*Subtotal*		207		143		152		1084		289		530		
*Total*														2405

## 3 RESULTS

### 3.1 Harbor seal sex ratio

Of the 2405 scat samples from the 13 selected haul‐out sites, 933 (38.8%) samples were confirmed to come from females, 948 (39.4%) from males, and the remaining 524 (21.8%) were undefined (failure rate of 21.8%). Overall, the sex ratio was 1:1.02 (female:male) but showed notable spatiotemporal variation (Table 1). Baby Island, Belle Chain, and Gertrude Island were “well‐sampled” (i.e., they had >100 samples) and had close to 1:1 sex ratios (Table 2). Out of the other well‐sampled haul‐out sites, Commencement Bay, Cowichan Bay, Cutts Area, and Fraser River had about two to three times as many males as females (Table 2). Comparatively, Nisqually and Woodard Bay were also well‐sampled and had about two and five times as many females as males, respectively (Table 2). Comparison of GLMMs indicated that haul‐out site was a useful predictor of variation in harbor seal sex ratios (Table 3). Haul‐out site explained about 16% of the variation in sex ratio, while adding year and season to the model only explained an additional 1% of the variation in sex ratio (Table 3). Multi‐year sampling revealed consistent sex ratios over time at male‐skewed sites like Fraser River and female‐skewed sites like Woodard Bay (Tables 1 and 2).

**TABLE 2:** Number of harbor seal scat samples collected in the Salish Sea at 13 haul‐out sites relative to sex.

Haul‐out site	Male	Female	Total
Baby Island	74	76	150
Belle Chain	73	62	135
Commencement Bay	123	56	179
Cowichan Bay	88	44	132
Cutts Area	87	43	130
Eagle Island	39	37	76
Fraser River	164	51	215
Gertrude Island	156	196	352
Nisqually	56	106	162
Penn Cove Mussel Farm	29	13	42
Port Gamble	1	25	26
Seal Rocks	16	17	33
Woodard Bay	42	207	249

**TABLE 3:** General Linear Mixed Models (GLMMs) of the influence of haul‐out site, season, and year on the sex ratio of harbor seals in the Salish Sea.

Model	DF	AIC	ΔAIC	BIC	$R^{2}m$	$R^{2}c$
**Haul‐out Site** + Year + Season	15	2335.14	0	2418.23	.16	.17
Year + Season	3	2517.26	182.12	2533.87	0	.05

*Note*: Fixed effects are labeled in bold. $R^{2}m$ denotes marginal $R^{2}$ values, and $R^{2}c$ indicates conditional $R^{2}$ values.

### 3.2 Sex‐specific harbor seal diet at the order level

**FIGURE 2:**
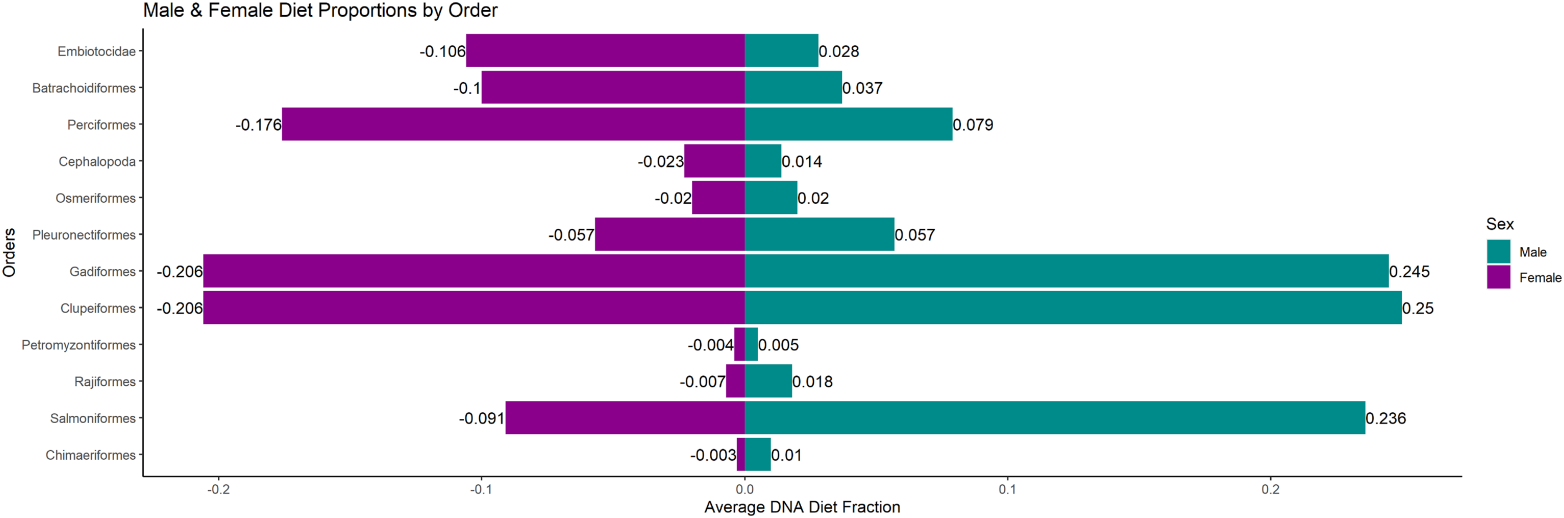
DNA male and female harbor seal diet fractions in the Salish Sea as average Relative Read Abundance (RRA) of each prey order. Only average DNA diet fractions ≥0.001 were shown to reduce the presence of minor taxa.

**FIGURE 3:**
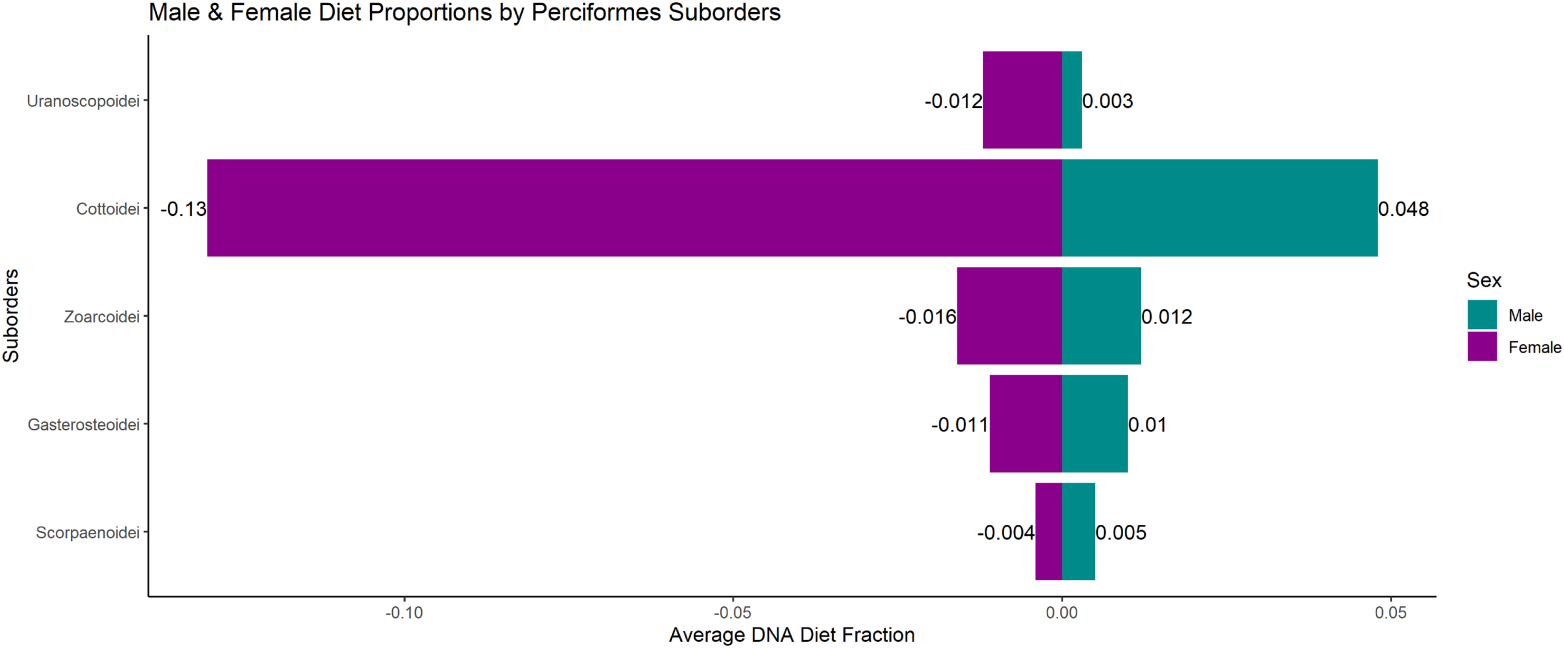
DNA male and female harbor seal diet fractions in the Salish Sea as average Relative Read Abundance (RRA) of each prey suborder within Perciformes.

We report averages of the proportions of reads assigned to different prey taxa within scat samples to characterize relative differences in diet between males and females. Clupeiformes made up a large proportion of relative male (25.0%) and female (20.6%) diet, followed closely by Gadiformes in male (24.5%) and female (20.6%) diet (Figure 2). Although Clupeiformes and Gadiformes did not differ much in the average diet of males and females, Salmoniformes were about 2.6 times more abundant in the diet of males (23.6%) than females (9.1%) (Figure 2). Conversely, Perciformes (17.6%), Embiotocidae (10.6%), and Batrachoidiformes (10.0%) were more abundant in the diet of females than males (Figure 2). Further, the majority of the Perciformes suborders comprised more of the average diet of females than males. Cottoidei, in particular, represented about 2.7 times more of the average female diet (13%) than male diet (4.8%) (Figure 3).

A PERMANOVA of the 10 prey orders with a mean RRA > 0.01 revealed that sex was a significant factor explaining diet differences overall, although its effect was small ($R^{2}=1.3\%,p<.05$) (Table 4). PERMANOVAs for the early season showed that sex and haul‐out site were both significant predictors of variation in diet during the early season, while the interaction between sex and haul‐out site was not significant (PERMANOVA: $R^{2}=1.7\%,p<.05$; $R^{2}=26\%,p<.001;R^{2}=4.7\%,p=.961,\text{respectively}$) (Table 4). PERMANOVAs filtering by the late season indicated that sex, haul‐out site, and the interaction between sex and haul‐out site were significant (PERMANOVA: $R^{2}=2.3\%,p<.05;R^{2}=54\%,p<.001;R^{2}=14\%,p=.008,\text{respectively}$; Table 4). Haul‐out site, as a factor from both the early and late season data, yielded the highest $R^{2}$ values, explaining the most dietary variation compared to other factors tested in each PERMANOVA. None of the tests for overdispersion were significant except for the factor combinations involving haul‐out sites in the early season subset (Table 4). Visual comparison of the data in an NMDS plot (Figure S1) suggests that differences in dispersion are likely caused by differences in sample size, with haul‐out sites with fewer samples being less dispersed than haul‐out sites with larger sample sizes. In such cases, the PERMANOVA tends to be overly conservative compared to a balanced sampling design (Anderson & Walsh, 2013).

**TABLE 4:** PERMANOVA results using the ten prey orders with an average diet proportion across the whole dataset of >0.01 filtered by early/late season.

	DF	Sums of Sqs	$R^{2}$	*F*	*p*(>*F*)
Early season					
Sex	1	0.585	.01717	2.3764	.035
Haul‐out site*	12	8.899	.26133	3.6853	.001
Sex × Haul‐out site*	11	1.592	.04675	0.7085	.961
Late season					
Sex	1	0.3673	.02341	1.3662	.251
Haul‐out site	11	8.4242	.5369	4.9536	.001
Sex × Haul‐out site	10	2.2443	.14303	1.7157	.008
Early + late season					
Sex	1	0.701	.01335	2.639	.019

*Note*: Factors labeled with an asterisk were found to be significant according to the betadisper function in R package vegan and may be overdispersed.

### 3.3 Sex‐specific harbor seal diet at the species level

**FIGURE 4:**
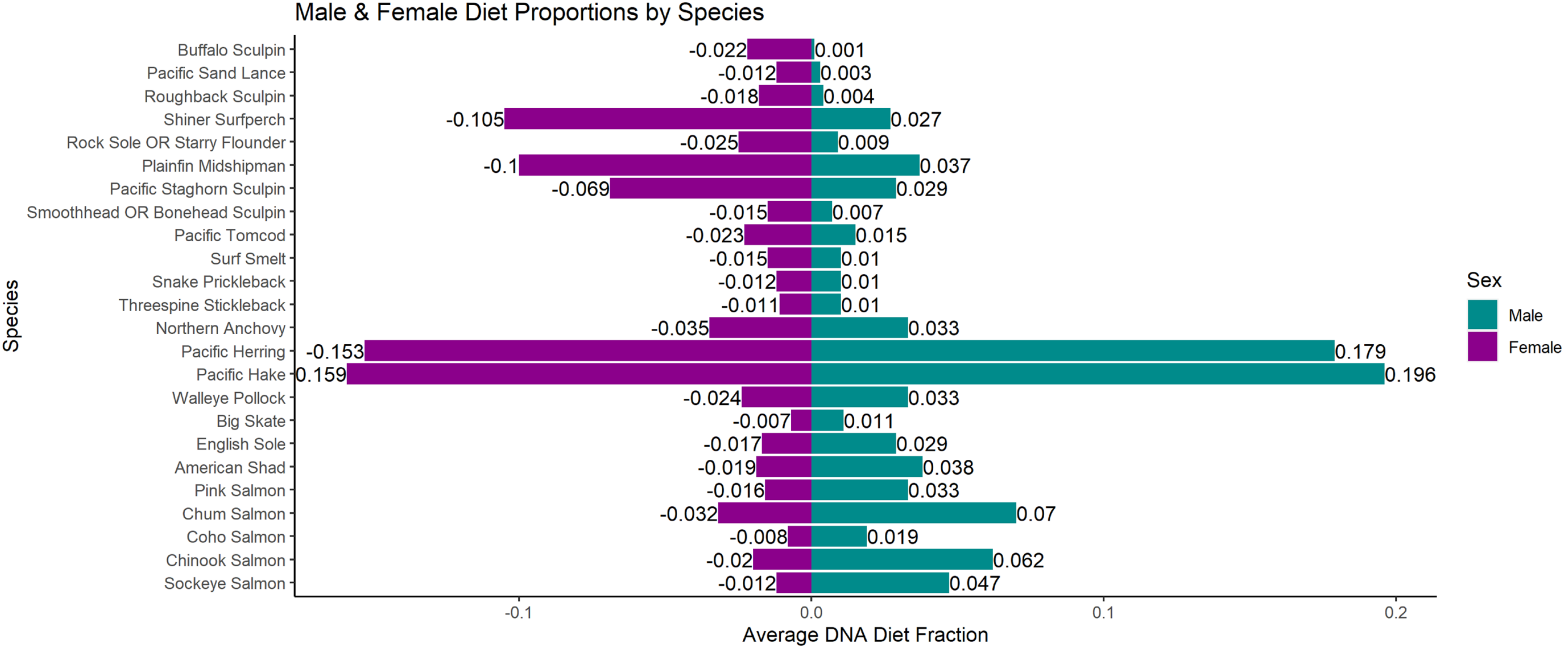
DNA male and female harbor seal diet fractions in the Salish Sea as average Relative Read Abundance (RRA) of each prey species. Only average DNA diet fractions ≥0.008 were shown to reduce the presence of minor taxa.

Pacific hake (*Merluccius productus*) and Pacific herring (*Clupea pallasii*) dominated male and female harbor seal diet, driving the prevalence of Clupeiformes and Gadiformes in the diet at the order level. Pacific hake comprised 19.6% of the average male's diet and 15.9% of the average female's diet (Figure 4). Pacific herring made up 17.9% of the average male's diet and 15.3% of the average female's diet (Figure 4). While Pacific herring and Pacific hake dominated total prey consumption, salmon species had stark differences in male and female harbor seal diet composition (Figure 4). Salmon species comprised far more of the average male's diet than the average female's diet (Figure 4). Shiner surfperch (*Cymatogaster aggregata*) (10.5%), plainfin midshipman (*Porichthys notatus*) (10.0%), and Pacific staghorn sculpin (*Leptocottus armatus*) (6.9%) additionally made up a large portion of the average female's diet (Figure 4).

### 3.4 Sex‐specific harbor seal diet with a focus on salmon

Out of five species of Pacific salmon—Chinook, chum, coho, pink (*Oncorhynchus gorbuscha*), and sockeye—Chinook salmon comprised ca. three times more and chum salmon ca. two times more of the average male harbor seal's diet than the average female's diet (Figure 4). This male bias for salmon was also observed in the remaining three salmon species that had lower average dietary proportions. Sockeye salmon comprised about four times more of the average male harbor seal's diet than the average female's diet (Figure 4). Coho and pink salmon showed less of a male bias in terms of average male diet, with both species making up ca. two times more than the average female's diet (Figure 4).

**FIGURE 5:**
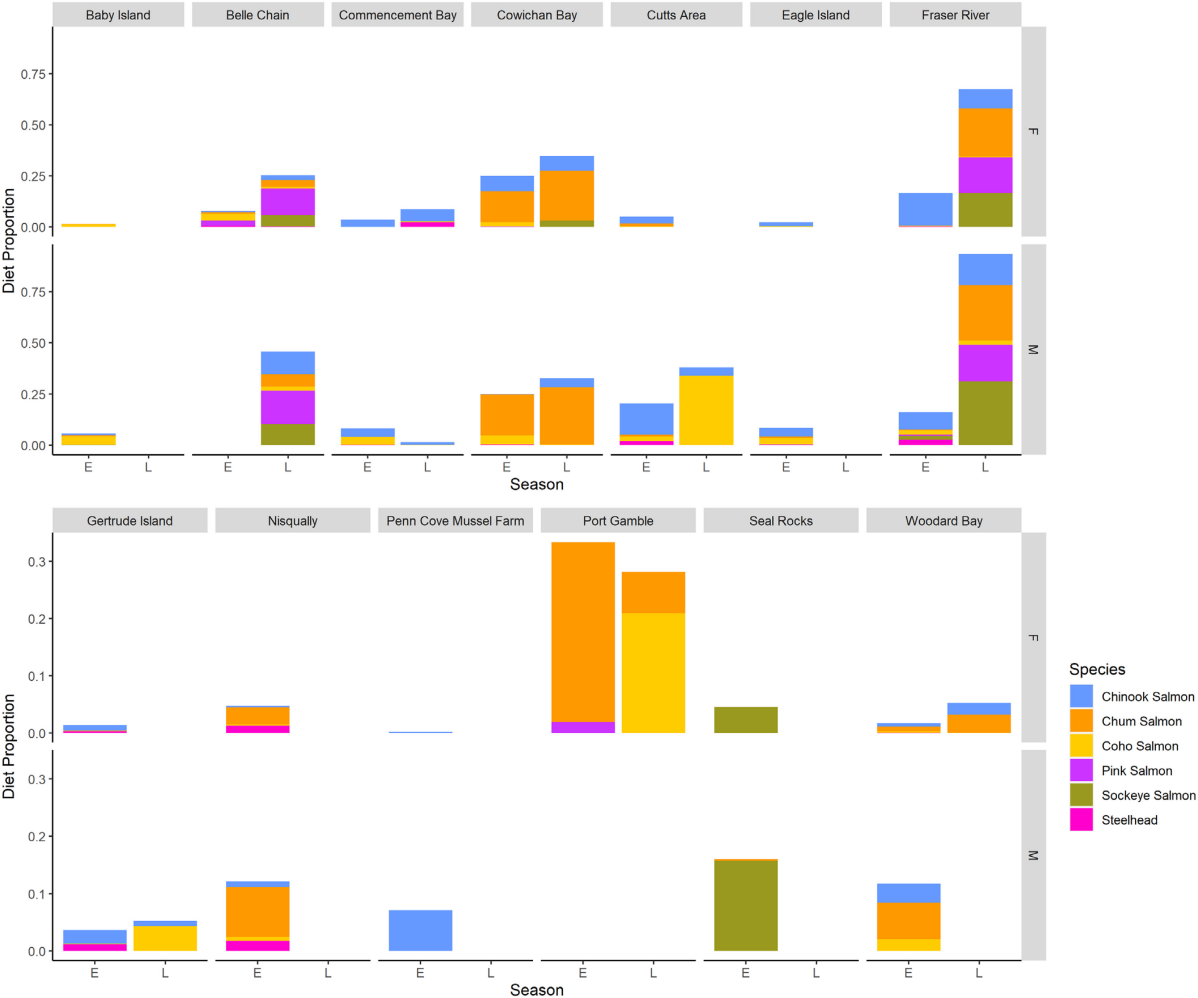
Salmon (*Oncorhynchus* spp.) consumption by male and female harbor seals at all haul‐out sites during the early (E) and late (L) seasons.

The degree of observed sex‐specific predation on salmon varied between the sampled haul‐out sites and across seasons. Across all 13 haul‐out sites, there appeared to be a slight overall male bias for salmon species (Figure 5). The vast majority of salmon consumption by both males and females also seemed to occur in the late season (Figure 5). Additionally, the overall dietary proportion of salmon at each haul‐out site was uneven, which was exemplified by a very large salmon proportion in the seal diet at Fraser River during the late season (Figure 5). The makeup of salmon species in harbor seal diet also varied between haul‐out sites (Figure 5). For instance, Cowichan Bay heavily featured chum, while haul‐out sites like Belle Chain and Fraser River shared more variety with all salmon species present (Figure 5).

### 3.5 Sex‐specific harbor seal diet by haul‐out site

**FIGURE 6:**
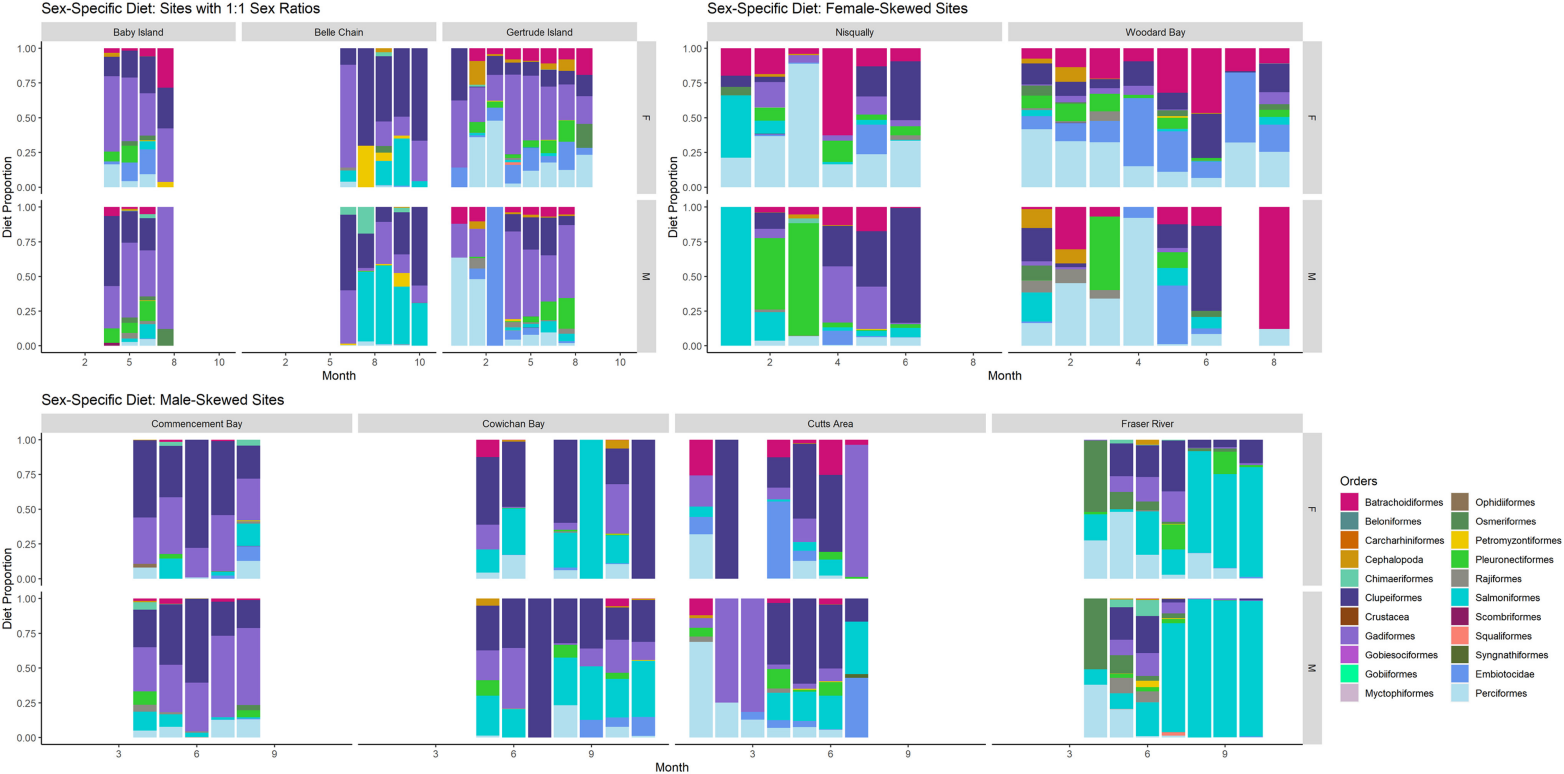
Male and female harbor seal diet composition in the Salish Sea by order at haul‐out sites relative to sex ratio: 1:1 sex ratio, male‐skewed sex ratio, and female‐skewed sex ratio.

As previously mentioned, Baby Island, Belle Chain, and Gertrude Island all had about 1:1 sex ratios (female:male) and were well‐sampled. Since these haul‐out sites had comparable numbers of males and females, it is possible to compare overall male and female diet between and within these haul‐out sites. In contrast, the dietary makeup of males and females at Gertrude Island varied drastically relative to that at Baby Island and Belle Chain (Figure 6). However, diets of males and females at each of these haul‐out sites was mostly similar, with only a couple of key outliers. While males ate more Salmoniformes at Belle Chain, females consumed more Perciformes, Batrachoidiformes, Pleuronectiformes, and Embiotocidae at Baby Island and Gertrude Island (Figure 6).

The starkest differences in diet were found between the male‐skewed and female‐skewed haul‐out sites. Male diet at the male‐skewed haul‐out sites was mainly composed of Clupeiformes, Gadiformes, and Salmoniformes, while female diet at the female‐skewed haul‐out sites was mainly made up of Batrachoidiformes, Embiotocidae, and Perciformes (Figure 6). Commencement Bay, Cowichan Bay, Cutts Area, and Fraser River were all well‐sampled and were characterized by sex ratios skewed toward males. As there were fewer female samples present for these haul‐out sites, the sex‐specific dietary analysis focused on the male diet. Male diet was comparable across these four haul‐out sites, with a large emphasis on Clupeiformes, Gadiformes, and Salmoniformes (Figure 6). Perciformes were also featured heavily in the male diet at Cutts Area (Figure 6). Fraser River stands out, with Salmoniformes the most dominant in male diet (Figure 6). Although females were the minority sex at these haul‐out sites, the female diet appeared to follow male diet (Figure 6).

In contrast to the above four sites, Nisqually and Woodard Bay had sex ratios skewed toward females (both well‐sampled). Thus, the sex‐specific dietary analysis focused on the female diet at these two haul‐out sites. Female diet at these sites was strikingly similar, mostly composed of Clupeiformes, Batrachoidiformes, Embiotocidae, and Perciformes (Figure 6). Males were the minority sex at these haul‐out sites, though they had similar diets to females, aside from more Pleuronectiformes, Clupeiformes, Gadiformes, and Salmoniformes featured in the male diet at Nisqually (Figure 6).

The male and female diets at the three haul‐out sites with about 1:1 sex ratios (female:male) showed some key differences from diets at the male‐skewed and female‐skewed haul‐out sites. Male diet at Gertrude Island varied drastically from male diet at the male‐skewed haul‐out sites due to a large dietary proportion of Perciformes at Gertrude Island (Figure 6). The strong emphasis on Salmoniformes at Fraser River also differed from Gertrude Island and Baby Island but more closely resembled male diet at Belle Chain (Figure 6). Conversely, female diet at Gertrude Island was similar to female diet at the female‐skewed haul‐out sites, except with a stronger representation of Clupeiformes and Gadiformes at Gertrude Island (Figure 6). Female diet at Baby Island and Belle Chain, however, differed greatly from the female‐skewed haul‐out sites, with more Clupeiformes, Gadiformes, and Salmoniformes present (Figure 6).

A comparison of LMMs examined the predictors of variation in Shannon diversity indices calculated with order‐level diet data from the well‐sampled haul‐out sites. The model excluding fixed effects (sex, haul‐out site, and haul‐out site type) was the best fit, indicating that the fixed effects were poor overall in predicting variation in Shannon diversity indices (Table 5). Even though the model included only the random effects (year and season), it was the best‐fit model, and it explained only a small fraction of the variation (Table 5).

**TABLE 5:** Linear mixed effects models (LMMs) of the influence of sex, haul‐out site, haul‐out site type (1:1, male‐skewed, or female‐skewed), season, and year on Shannon Diversity Indices generated from all 22 prey orders.

Model	DF	AIC	ΔAIC	BIC	$R^{2}m$	$R^{2}c$
Year + Season	4	262.33	0	274.55	0	.11
**Sex +** Year + Season	5	266.43	4.1	281.71	0	.11
**Site Type +** Year + Season	6	266.44	4.11	284.78	.03	.11
**Sex + Site Type +** Year + Season	7	270.72	8.39	292.12	.04	.15
**Haul‐out Site +** Year + Season	12	281.51	19.18	318.18	.06	.16
**Site + Site Type +** Year + Season	12	281.51	19.18	318.18	.06	.16
**Sex + Haul‐out Site +** Year + Season	13	285.9	23.57	325.63	.07	.17
Sex + Haul‐out Site + Site Type (Sex*Haul‐out Site*Site Type) + Year + Season	21	296.74	34.41	360.92	.11	.21

*Note*: Fixed effects are labeled in bold. $R^{2}m$ denotes marginal $R^{2}$ values, and $R^{2}c$ indicates conditional $R^{2}$ values.

## 4 DISCUSSION

### 4.1 Sex and haul‐out site‐specific harbor seal diet discrepancies and potential causes

Our study presented data on sex ratios and diets of harbor seals across the Salish Sea from 2012 to 2018 (Thomas et al., 2022; Voelker et al., 2020). To our knowledge, this is the most spatiotemporally diverse look at the sex‐specific diet of harbor seals in the Salish Sea region to date. Our analysis showed that while there were virtually even proportions of male and female harbor seals in the Salish Sea overall, each sex was respectively more highly concentrated within particular haul‐out sites and regions. Between 40% and 50% of the mean diet of both males and females was composed of Clupeiformes and Gadiformes, and sex‐specific differences were found in the orders making up the remaining diet. The main differences between the relative diet of males and females were a male dietary bias for Salmoniformes and a female dietary bias for Perciformes, Embiotocidae, and Batrachoidiformes (Figure 2). With the second‐highest Salmoniform dietary proportion, Chinook salmon composed significantly more of the average male harbor seal's diet than the average female's diet (Figure 4). Additionally, the male diet at haul‐out sites with male‐skewed sex ratios emphasized Clupeiformes, Gadiformes, and Salmoniformes, while female diet at female‐skewed haul‐out sites was mostly composed of Clupeiformes, Batrachoidiformes, Embiotocidae, and Perciformes. These data suggest marked dietary differences between male and female harbor seals in the Salish Sea, with a notable male bias for salmon and varying diets and sex ratios specific to haul‐out sites.

One of the biggest deviations from the more limited study by Schwarz et al. (2018) was the absence of any noticeable differences in diet diversity between the sexes in our current study (Table 5). The type of haul‐out site (1:1, male‐skewed and female‐skewed) and haul‐out site itself also did not strongly affect diet diversity (Table 5). One potential explanation may be the greater ecological diversity of haul‐out sites in our study compared to the two estuarine sites within closer proximity in Schwarz et al. (2018). The fact that Schwarz et al. (2018) found such a strong sex effect on Shannon diversity indices in contrast to our study should caution those attempting to extrapolate region‐wide harbor seal diet patterns from a small sample base.

The haul‐out site appeared to be the factor most influencing the dietary differences detected between males and females, with these dietary differences resulting from sex‐specific spatial assortment. Males and females appeared to be consuming similar selections of prey at each haul‐out site, but their differing dietary biases were consistent across the Salish Sea. Given that male and female diets from haul‐out sites with even sex ratios mostly featured the same prey items, it is likely that males and females were both mainly eating a localized selection of prey items that varied from haul‐out site to haul‐out site. Sex‐specific diet differences mainly appeared to be associated with haul‐out sites, with a “male diet” being dominated by males and haul‐outs with a “female diet” dominated by females. There was also, however, a haul‐out‐independent sex‐specific effect. At a haul‐out site with an even sex ratio like Belle Chain, male and female diet mainly consisted of Clupeiformes, Gadiformes, and Salmoniformes, yet the male diet still more heavily featured Salmoniformes than the female diet (Figure 6). Further, both males and females consumed Batrachoidiformes and Perciformes at Gertrude Island (another haul‐out site with an even sex ratio), but the female diet was still composed of more Batrachoidiformes and Perciformes than the male diet (Figure 6).

Comparison of male diet at male‐skewed haul‐out sites and female diet at female‐skewed haul‐out sites highlighted these dietary differences. Female‐skewed haul‐out sites were characterized by large female dietary proportions of Perciformes, Batrachoidiformes, and Embiotocidae, corresponding to the abundance of sculpins (Cottoidei), plainfin midshipman, and shiner surfperch in the average female diet (Figure 4). Other harbor seal diet studies in the Salish Sea have also reported shiner surfperch, plainfin midshipman, and sculpins as a key part of harbor seal diet (e.g., Bjorkland et al., 2015; Lance et al., 2012; Thomas et al., 2022). This female bias for benthic and estuarine prey species has been previously reported (Schwarz et al., 2018) and was already suspected by the finding that small females in Puget Sound and the Strait of Juan De Fuca had isotopic values close to that of the nearshore environment (Bjorkland et al., 2015). Previous studies have also found that females tend to undertake deeper dives than males, which may explain the abundance of benthic species found in female diet (Wilson et al., 2014). Females dominated the sex ratio at Nisqually and Woodard Bay, which are protected inlets. This finding may indicate that females are using these haul‐out sites as pupping haul‐out sites and are performing deeper dives near their pupping haul‐out sites due to reproductive, energetic restrictions rather than traveling outward for different prey (Bjorkland et al., 2015; Schwarz et al., 2018). Female harbor seals in the Salish Sea appear to move shorter distances than males, supporting the idea that females forage near their haul‐out sites (Peterson et al., 2012).

Consistent with Schwarz et al. (2018), overall male diet shared the two pelagic fish species Pacific herring and Pacific hake with females, while showing a higher proportion of pelagic salmon and lower proportions of benthic species than the female diet. Previous harbor seal diet studies in the Salish Sea have indicated the importance of Pacific herring, Pacific hake, and salmon in overall harbor seal diet and distribution (e.g. Bjorkland et al., 2015; Lance et al., 2012; Thomas et al., 2022). However, our observed male dietary bias for salmon may be specific to the Salish Sea region. For example, in contrast, a recent study in Japan found a female dietary bias for salmon instead (Jimbo et al., 2021). In the Salish Sea, the male diet at male‐skewed haul‐out sites was comprised more of these pelagic species than female diet at female‐skewed haul‐out sites. This begs the question of whether males concentrate at certain haul‐outs for reasons unrelated to foraging and simply happen to be in the vicinity of their “preferred” prey or are seeking out this type of prey and therefore utilize haul‐outs close to their preferred prey. Although the number of male‐dominated haul‐out sites is small, it is noteworthy that all show high salmonid, gadid, and clupeid diet proportions, while none of the male‐dominated haul‐outs show large proportions of “female” preferred prey. This pattern appears to be consistent with the notion of males choosing certain haul‐outs for diet‐related reasons. Regardless, the marked diet differences between sites with oppositely skewed sex ratios highlights the probable impact of sex‐specific spatial assortment on harbor seal diet. Male harbor seals additionally do not provide parental care and are known to travel further distances in the Salish Sea than female harbor seals, which are spatially restricted during pupping season (Peterson et al., 2012; Schwarz et al., 2018). While some sex‐specific diet differences can be explained by varying body size between males and females, harbor seals do not have prominent sexual dimorphism (Schwarz et al., 2018). This points toward sex‐specific diet differences arising from other factors, such as female harbor seal reproductive costs and differing movement patterns between males and females (Peterson et al., 2012; Schwarz et al., 2018). Diet differences have additionally been found between harbor seals of varying age classes. One study based in the Salish Sea found that subadult harbor seals of both sexes consumed the greatest proportion of biomass, followed by adult females, adult males, and pups of both sexes (Howard et al., 2013). Male harbor seals also restrict their foraging range during weaning to mate with females, potentially resulting in lower diet diversity throughout this period (Schwarz et al., 2018).

### 4.2 Predation pressure and management implications

All four male‐skewed haul‐out sites are located near currently active salmonid hatcheries (Figure 6). The Fraser River haul‐out site is within about 40 km of four hatcheries, with the closest hatcheries supporting runs of Chinook, chum, and coho salmon (Periscopic & Pacific Salmon Foundation Salmon Watersheds Program, 2022). Two hatcheries sit near Cowichan Bay, the nearest being approximately 4 km away and assisting with Chinook, chum, and coho runs (Periscopic & Pacific Salmon Foundation Salmon Watersheds Program, 2022). Cutts Area is located within 6 km of a hatchery supporting Chinook and coho salmon runs (WDFW SalmonScape, 2004). Commencement Bay is about 15 miles away from a hatchery that produces Chinook and coho salmon (WDFW SalmonScape, 2004). In fact, three of the four male‐skewed haul‐out sites sampled showed a male bias for salmon and may be considered predation hotspots: Cowichan Bay, Cutts Area, and Fraser River. Of the five Pacific salmon species consumed, Chinook salmon was the only species found in the male harbor seal diet at all three potential predation hotspots. However, ultimately determining a foraging hotspot to target for management should depend on the salmon behavioral response and whether salmon are decreasing at a site. The status of salmon at Cowichan Bay, Cutts Area, and Fraser River should be compounded with the male‐skewed sex ratios and male dietary biases for salmon to indicate if male harbor seals are asserting undue predation pressure warranting targeted management at these haul‐out sites.

We found that while males consume higher proportions of salmon, females consume predators of juvenile salmon such as Pacific staghorn sculpin (Mace, 1983) and other sculpins (Berejikian, 1995; Tabor et al., 1998). This echoes a previous hypothesis by Schwarz et al. (2018) that female harbor seal predation on salmonid predators may result in indirect positive effects on salmon abundance. This potential scenario poses issues for management, since male and female harbor seals may have opposing effects on threatened salmon at certain haul‐out sites. Male and female predation by harbor seals may even out in the Salish Sea as a whole and at haul‐out sites with a sex ratio closer to 1:1 (female:male). However, local salmon runs at haul‐out sites that are male‐skewed or female‐skewed may experience different levels and types of impact on salmonids. The overall impact of seals on salmon may be more complicated at female‐skewed haul‐out sites (e.g., Nisqually and Woodard Bay), where the diet contained many salmon predators. On the other hand, as described before, predation pressure on salmon at male‐skewed haul‐out sites (e.g., Fraser River) could be more intense than at female‐skewed sites, given that relative male diet there was primarily composed of salmon. Further biomass and ecosystem‐based modeling is necessary to fully understand the impacts of harbor seal predation on salmon species.

Although our data suggests that the male harbor seal diet contains more salmon than the female harbor seal diet, we preface this finding by noting that the actual quantitative impact of males on salmon at any given haul‐out site in the Salish Sea likely varies based on the sex ratio at that haul‐out site as well as the number of males and the abundance/density of salmon. Male harbor seals may be exerting strong predation pressure on salmon at the aforementioned “predation hotspots,” but male harbor seals found at female‐skewed haul‐out sites or at haul‐out sites with an even sex ratio may not be having much of an impact on salmon. Further, male diet at a few haul‐out sites with a male presence (either even or male‐skewed sex ratios), like Gertrude Island, Baby Island, and Commencement Bay, actually contained mostly prey taxa other than salmon, suggesting that male harbor seal predation on its own may not be of conservation concern. However, to answer this question, we would need to examine the response of the salmon population of interest to such male harbor seal predation. For sites in which local salmon populations are declining, we suggest that management focuses on male‐skewed haul‐out sites with a demonstrated male harbor seal bias for salmon.

### 4.3 Study caveats

The unbalanced sampling design used in this study limited the extent of analysis. Due to opportunistic sampling throughout space and time, we were unable to fully decouple temporal from spatial variation, although patterns seem consistent. Gaps in sampling additionally complicated data analysis, and we were unable to test certain factors (i.e., year and season). Samples were also mainly collected in either the early or late seasons, thus inherently biasing results to these periods, and no site was sampled year‐round. Much of the sampling also occurred during pupping season, which generally ranges from June to September at all represented haul‐out sites in the Salish Sea (Huber et al., 2001; Jeffries et al., 2000). Roughly 14% of all scat samples from the 13 haul‐out sites in this study were collected from females during June through September. Since harbor seal mothers tend to forage with their pups near their haul‐out site instead of venturing outward, diet differences between sexes may have been inflated during pupping season (Schwarz et al., 2018). To address limitations in quantifying sex‐specific diet, diet differences are described as proportions of barcoding reads. These are likely biased estimates of actual diet due to factors such as potential biases in sequence recovery (Deagle et al., 2018). However, there is no unbiased method of describing diet (Bowen & Iverson, 2012; Deagle et al., 2018; Sousa et al., 2019). Our study focused on the relative diet differences between sexes in time and space and did not seek to provide absolute diet estimates. We also used diet data from Thomas et al. (2022) and followed the taxonomic assignment of species in that study. Therefore, in cases where Thomas et al. (2022) were ambiguous, that same ambiguity was present in our diet data. We were additionally unable to compare the proportions of smolt salmon versus adult salmon in male and female diet. While Schwarz et al. (2018) was able to distinguish adult from juvenile salmon by combining DNA and hard parts data, we did not have access to hard parts data. Due to the aforementioned temporally uneven sampling, we also decided not to assign a salmon life stage using season alone to avoid decoupling season from the haul‐out site. Additionally, we were only able to examine the relative proportions of salmon in harbor seal diet rather than the response of salmon to harbor seal predation.

## 5 CONCLUSIONS

Our analysis highlights the variation in male and female harbor seal distribution and diet at a relatively large scale within the Salish Sea (ca. 18,000 km^2^; The Salish Sea & Surrounding Basin, 2023). Diet differences between males and females appear to be largely related to differences in sex‐specific spatial and geographical assortment combined with opportunistic use of local prey resources and geographical assortment of prey. The previously reported male harbor seal bias for salmon (Schwarz et al., 2018) appears to extend across the Salish Sea, and salmon runs at male‐dominated haul‐out sites such as Cowichan Bay, Cutts Area, and Fraser River may experience disproportionate predation pressure. These haul‐out sites may require more attention from management, namely increased monitoring of harbor seal predation and of salmon population response to such predation.

## AUTHOR CONTRIBUTIONS

**Holland C. Conwell:** Data curation (equal); formal analysis (lead); investigation (supporting); methodology (equal); validation (equal); visualization (lead); writing – original draft (lead); writing – review and editing (lead). **Zoë K. Lewis:** Data curation (equal); investigation (lead); methodology (equal); writing – review and editing (supporting). **Austen Thomas:** Conceptualization (equal); funding acquisition (equal); methodology (equal); writing – review and editing (supporting). **Alejandro Acevedo‐Gutiérrez:** Conceptualization (equal); funding acquisition (equal); methodology (equal); project administration (equal); supervision (equal); writing – review and editing (equal). **Dietmar Schwarz:** Conceptualization (equal); formal analysis (supporting); funding acquisition (equal); methodology (equal); project administration (equal); supervision (equal); validation (equal); writing – review and editing (equal).

## Supporting information


Data S1

Data S2

Figure S1

## Data Availability

The diet study data used is publicly available at https://doi.org/10.1038/s41597‐022‐01152‐5. This includes the proportion of each prey species within each sample as determined by metabarcoding and hard part analysis. The diet data corresponding to sex and sample ID are included within Supplementary Files and organized by order and species.
