# Supplementary material for: Sex‐specific diet differences in harbor seals (Phoca vitulina) via spatial assortment
Source: Ecol Evol. 2024 Jul 3;14(7):e11417. doi: 10.1002/ece3.11417 (PMC11222013; doi:10.1002/ece3.11417)

**Supplementary Materials**

Supplemental Figure 1. NMDS plot of the early season data with the same variables (sex and haul-out site) that were tested for overdispersion, in which haul-out site was found to be significant (Table 4).


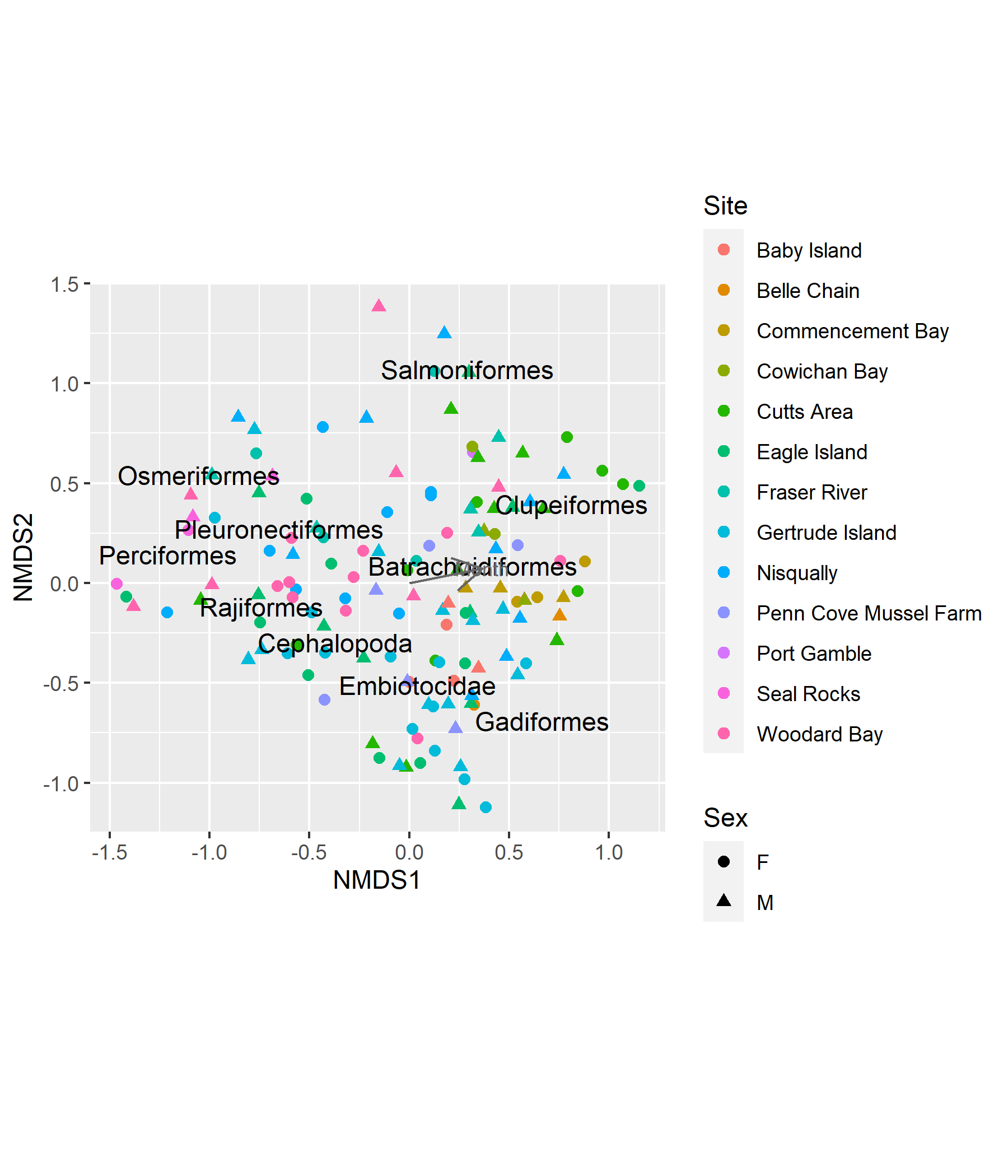

Supplement: Supplementary file 3 — Figure S1 [file ECE3-14-e11417-s003.docx]
